# Patient-specific implants for intraoral and maxillofacial reconstruction: a scoping review on customization and fabrication methods

**DOI:** 10.1186/s40902-025-00485-6

**Published:** 2025-10-07

**Authors:** Anjali G Pai, Shilpa S Prabhu

**Affiliations:** Department of Prosthodontics and Crown & Bridge, Manipal College of Dental Sciences, Manipal University, Manipal Academy of Higher Education, Manipal, India

**Keywords:** Patient specific implants (PSI), Maxillary defects, Mandibular defects, CAD-CAM,3D printing, Finite element analysis (FEA)

## Abstract

**Background:**

Maxillofacial defects compromise both function and aesthetics, posing significant challenges in rehabilitation. The advent of digital technologies has enabled the development of patient-specific implants (PSIs), providing individualized solutions that enhance clinical outcomes.

**Main body:**

This scoping review, conducted following the PRISMA-ScR protocol, explored literature published between January 2015 and January 2025 across PubMed, SCOPUS, Web of Science, and COCHRANE databases using the PCC framework. Eligible studies included original research, case reports, randomized and non-randomized trials, and finite element analyses addressing intraoral rehabilitation with PSIs. Evidence highlights that CAD/CAM and 3D printing facilitate the fabrication of anatomically precise, patient-matched implants. These technologies contribute to reduced surgical time, high implant survival, improved mastication, and enhanced speech outcomes, while complication rates remain low. Comparative findings suggest no major differences in fit or longevity between milled and printed PSIs. Furthermore, ongoing innovations such as bioprinting and tissue engineering offer potential pathways toward biologically integrated maxillofacial solutions.

**Conclusion:**

PSIs represent a promising modality in maxillofacial prosthetic rehabilitation. Optimizing outcomes requires continued research into advanced materials and digital fabrication techniques to expand their clinical scope.

**Supplementary Information:**

The online version contains supplementary material available at 10.1186/s40902-025-00485-6.

## Background

Ablative tumour surgery of the midface often results in significant aesthetic and functional impairments, imposing both physical and psychological burdens on patients [1]. Reconstruction of these complex defects remains challenging, with various techniques reported in the literature [2]. One of the reconstruction techniques includes the use of autogenous bone grafts, which are favored for their excellent biocompatibility; however, they are associated with potential risks such as donor-site morbidity and the possibility of surgical failure [3]. Implants avoid donor-site issues but may raise concerns about biocompatibility and cost. Moreover, conventional techniques are not tailored to the defect, often resulting in residual facial asymmetry [4]. Advancements in computer-aided design and manufacturing (CAD/CAM) technology have now enabled personalized treatment plans, even for complex facial skeletal cases, leading to improved surgical outcomes [5]. However, the diversity in design protocols, materials, and fabrication techniques creates a need to systematically map the existing landscape [6]. This scoping review aims to explore and summarize the current trends, technologies, and clinical considerations in the fabrication of customized implants for maxillofacial reconstruction, thereby identifying gaps in knowledge and guiding future research and clinical practice.

## Materials and methods

This scoping review protocol followed the guidelines by PRISMA-ScR (Preferred Reporting Items for Systematic Review and Meta-Analyses Protocols extension for Scoping Reviews)—Extension for Scoping Reviews. A comprehensive search was conducted across four electronic databases: PubMed, SCOPUS, Web of Science, and COCHRANE to identify relevant literature. Google Scholar was utilized to identify the grey literature and additional articles that may not be indexed in major electronic databases. The keywords used for the search included “patient-specific implant,” “intraoral defect,” “mandibular defects,” “maxillary defects,” “CAD-CAM,” and “3D printing.” A further manual search was performed, checking for eligible papers in the bibliographies of the initially retrieved articles and exploring the websites of relevant journals.

### Review question

The research was designed according to the Population, Concept, and Context (PCC) framework as follows:

- P: In patients with intraoral defects (both maxilla and mandible).
- C: Patient-Specific Implants (PSIs) manufactured using CAD-CAM and 3D printing.
- C: Dental clinical settings.

### Review question

The management of intraoral defects involving the maxilla and mandible remains a clinical challenge in dentistry. Advances in digital technologies, including CAD-CAM and 3D printing, have enabled the fabrication of patient-specific implants (PSIs) tailored to individual needs. However, the extent and the nature of their use in dental clinical practice are not well defined.

This scoping review aims to map the existing literature and explore how PSIs produced using CAD-CAM and 3D printing are utilized in managing intraoral defects. The review is guided by the following question, structured using the Population–Concept–Context (PCC) framework:

What is the role of patient-specific implants (PSIs) fabricated using CAD-CAM and 3D printing in the management of intraoral defects in dental clinical practice?

### Eligibility criteria

The review included studies published in English between January 2015 and January 2025 that focused on the rehabilitation of intraoral defects with patient-specific implants (PSIs). Eligible sources included full-text original research articles, clinical case reports, randomized and non-randomized controlled trials, and finite element analysis (FEA) studies.

Studies were excluded if they were animal studies, non-English publications, editorials or non-peer-reviewed opinion pieces, conference abstracts and proceedings, book chapters, or if they were not related to the use of PSIs for intraoral rehabilitation.

### Search strategy

Two authors independently performed all steps in the study selection and data extraction phase. Any disagreement between the two reviewers was resolved through discussion and consensus. The number of results retrieved from each database for this scoping review has been described in detail in Table 1.

**Table 1:** Number of results retrieved from each database

Sr No	Database	No of articles
1	PubMed	28
2	SCOPUS	336
3	Web of Science	45
4	COCHRANE	7
5	Google Scholar	229

The detailed search strategies for each electronic database and the websites of relevant journals have been provided in the Supplementary file 1.

### Data extraction

A standardized data extraction form was developed based on the objectives of our review and the Population–Concept–Context (PCC) framework. Details such as title, authorship, and publication year were extracted from each included study. Information on type of study, patient characteristics, and the type of reconstruction was also collected. Emphasis was placed on customization aspects, including materials, anatomical site, and design techniques. Surgical integration, complications, follow-up duration, and clinical outcomes were noted.

## Results

**Fig. 1:**
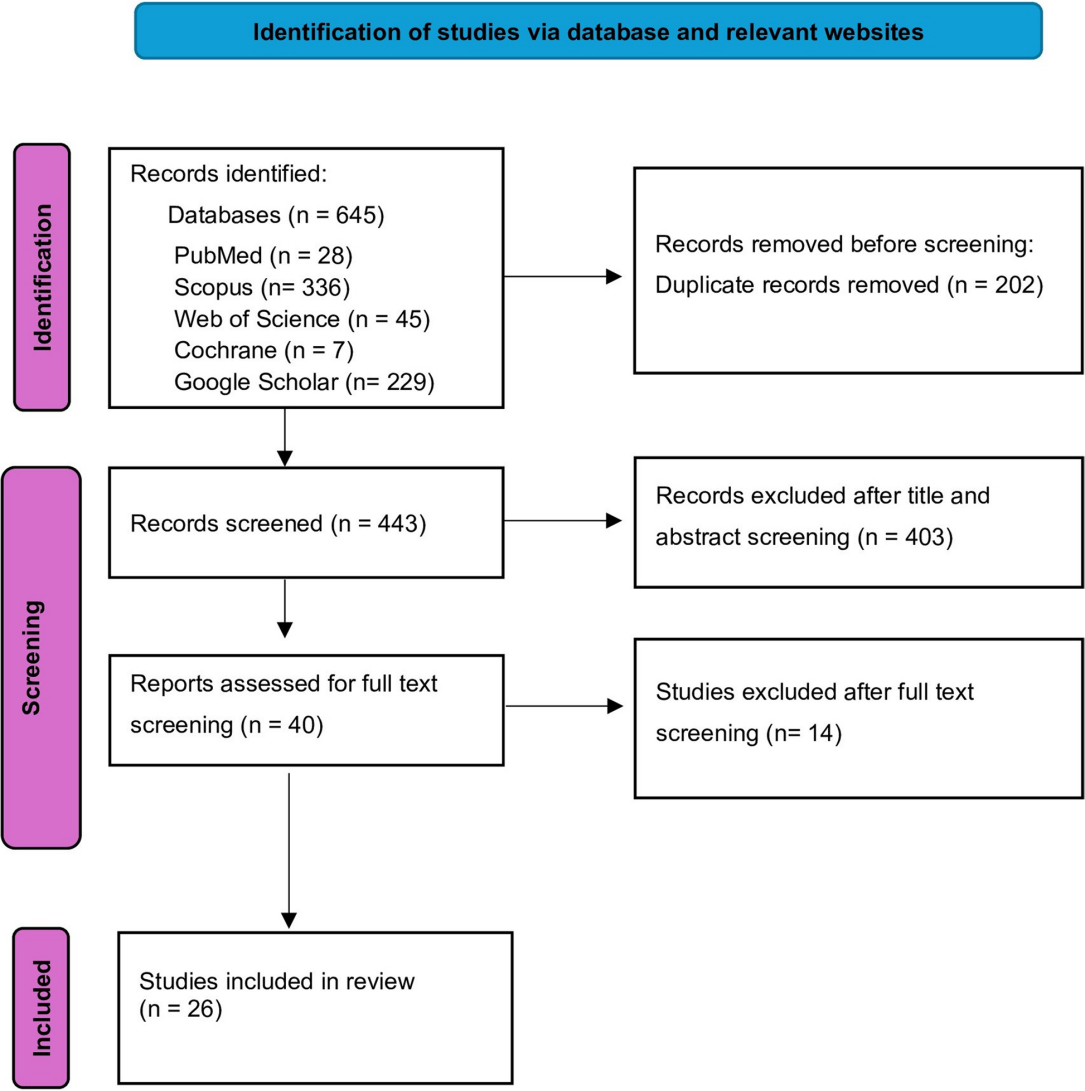
Flowchart of the study selection process

A total of 645 articles were initially identified through the scoping review process. After the removal of 202 duplicates, 443 articles remained for screening. Following full-text assessment, 417 studies were excluded based on the eligibility criteria, resulting in 26 studies being included in the final review (Fig. 1).

### Study characteristics

**Fig. 2:**
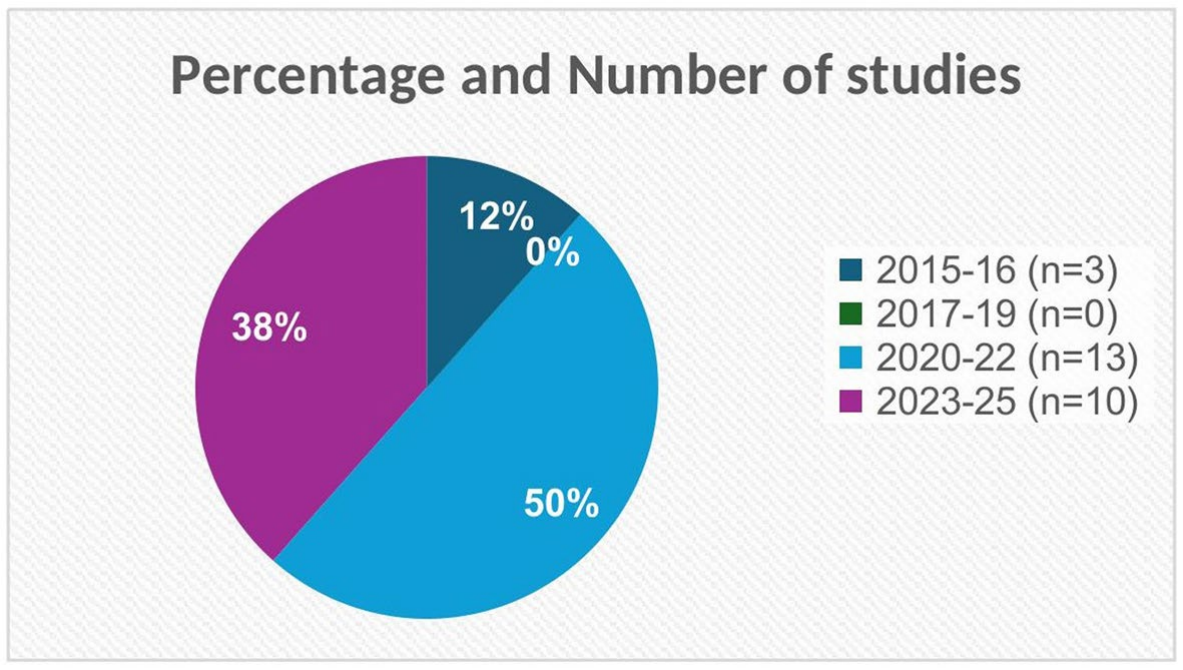
Percentage and number of studies by year of publication

The included studies were published between January 2015 and January 2025, with the majority appearing after 2019 (Fig. 2).

**Fig. 3:**
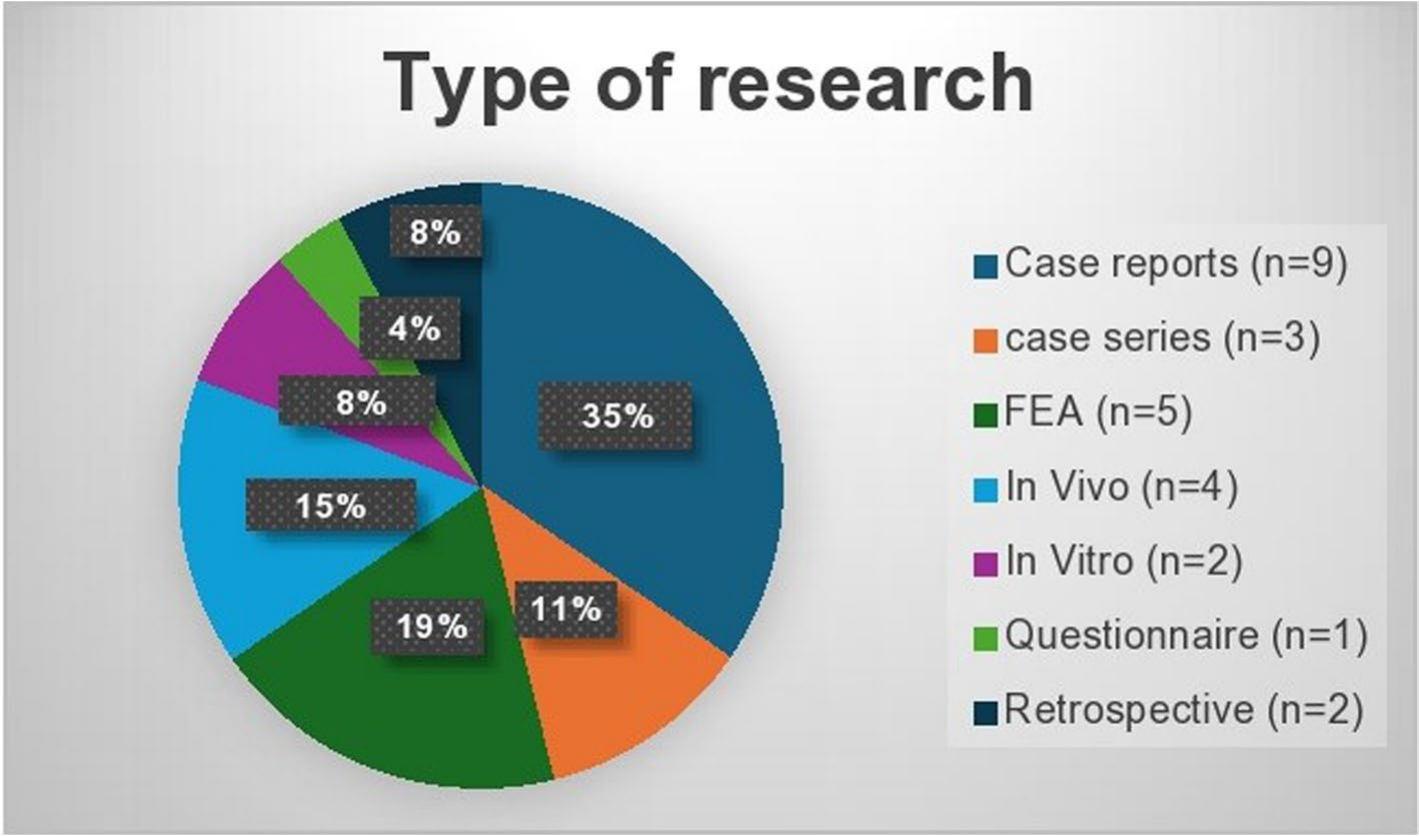
Number and percentage of studies by type of research

Case reports were the most reported study type (*n* = 9), followed by finite element analysis (FEA) studies (*n* = 5) and case series (*n* = 3). Fewer studies employed in vivo methodologies (*n* = 4) or preclinical approaches, including in vitro experiments (*n* = 2), a questionnaire-based study (*n* = 1), and retrospective analysis (*n* = 2) (Fig. 3).

Relevant data on patient-specific implant use, as reported in multiple articles, have been organized in Table 2. (The detailed dataset is provided in the Supplementary file 1: Table S2))

**Table 2:** Summary of included studies reporting on the use of customized, patient-specific implants for intraoral and maxillofacial reconstruction

Sr no:	Authors and Year	Study Type	Population and Intervention	Main Findings
1	Gugliotta Y, Zavattero E, Ramieri G, Borbon C, Gerbino G; 2024 [7]	Case series	**-Population:** 45 patients undergoing cranio-maxillofacial reconstruction (5 years post-resection) -**Intervention:** PEEK patient-specific implants Assessed morpho-functional outcomes (modified Katsuragy Scale), pain (VAS), and aesthetics (FACE-Q |Aesthetics)	• No prosthesis dislocation, rupture, or long-term infection • 12-month follow-up demonstrated favorable results • Complication: post operative infection was noted
2	Datarkar, Abhay N.; Pardiwala, Arwa F.; Relan, Priyanka; Daware, Surendra; Gadve, Vandana; Deshpande, Archana; Ghormade, Ashlesha; 2024 [8]	Finite element analysis	-**Population:** Patients with bilateral maxillectomy defects -**Intervention:** Patient-specific implants with strut abutments CT-based evaluation assessed physiological/pathological loading, implant failure and fatigue, osseointegration, and stress shielding of bone	• 100% osseointegration between the screws of the implant and the bone • Improved accuracy in stress distribution • Improved quality of life of patients noted
3	Arvind U.D, Gaddipati, R., Alwala, A.M.; 2024 [9]	In vivo	-**Population:** 10 patients undergoing surgical and prosthetic maxillary reconstruction -**Intervention:** Zygomatic implants and 3D-manufactured patient-specific implants At baseline, 6, and 12 months, assessments included pain, implant exposure, infection, wound dehiscence, implant fit, surgical rating, oro-antral communication, facial edema, and orbital status	• Efficient therapeutic outcomes for maxillary reconstruction • Good patient satisfaction • Although favourable outcomes reported, the sample size was small
4	Chakravarthy C, Patil RS, Wagdargi S, et al.,2024 [10]	Case report	-**Population:** Patient with segmental mandibular defect -**Intervention:** Customized computer-designed patient-specific implants (PSIs)	• CT and OPG evaluations over 2.5 years showed satisfactory aesthetics and favourable clinical outcomes
5	Alwala A.M., Ramesh, K., Swayampakula, H; 2023 [11]	In vivo study	-**Population:** 20 patients undergoing surgical and prosthetic maxillary reconstruction -**Intervention:** 3D-printed titanium subperiosteal implants	• At baseline, 3, 6, and 12 months, assessments included pain, implant exposure, infection, wound dehiscence, implant fit, surgical rating, and patient experience evaluation. One-year follow-up showed favorable outcomes, though limited by small sample size
6	Yang Z, Zhang J, Xu Z, Liu X, Yang J, Tan J; 2023 [12]	Finite Element Analysis	-**Population:** Patients with severe atrophic posterior maxilla -**Intervention:** Short patient-specific implants	• CBCT evaluation assessed implant position, structure, and wing spread area, demonstrating satisfactory anatomical adaptation
7	Manekar VS, Datarkar AN, Ghormode A, Daware S, Pandilwar P, Sapkal P.; 2023 [13]	Finite Element Analysis	**-Population:** Patient with post-COVID maxillary mucormycosis presenting unilateral/bilateral defects -**Intervention:** CAD/CAM patient-specific implants (PSI with struts and screw-retained) and quad zygoma implants	• CT-based evaluation validated and compared the biomechanical benefits of PSI designs (PSI 1, PSI 2) versus QZI, demonstrating favourable structural performance
8	Ayhan M, Cankaya AB.; 2023 [14]	Finite Element Analysis	-**Population:** Patients with insufficient bone tissue for conventional implant treatment -**Intervention:** 3D-customized subperiosteal implant designs (monoblock and dual implant systems)	• CT-based analysis calculated residual stress and displacement values on implant and jawbone models, showing biomechanical feasibility of both designs
9	Hwang B Y, Noh K, Lee JW., et al., 2023 [15]	Case report	-**Population:** Patient with osteosarcoma of the left mandibular body -**Intervention:** Segmental mandibulectomy followed by rehabilitation with osteocutaneous fibula free flap and 3D bioprinted PCL implant	• CT (2 weeks, annually for 6 years) and MRI (5, 11, 40 months, 4.5 years) demonstrated successful reconstruction outcomes. Limitations included high cost and time consumption
10	V jain, Vaibhav; Nagori, Shakeel.,2023 [16]	Case report	-**Population:** COVID patient with mucormycosis who underwent right orbital exenteration and maxillectomy -**Intervention:** 3D-printed patient-specific implant	• CT evaluation with follow-up up to 74 months demonstrated stable reconstruction outcomes
11	Simon Spalthoff S., Borrmann, M., Jehn, P.; 2022 [17]	In vitro study	-**Population/Procedure:** 25 digital datasets of temporary prostheses on virtually constructed edentulous maxillae -**Intervention:** Patient-specific maxillofacial implants	• CT evaluation assessed digital workflow efficiency, showing improved accuracy of prosthetic teeth positioning with the virtual design compared to the conventional method
12	Atef M Mounir M, Shawky M, Mounir S, Gibaly A, 2022 [18]	In vivo	-**Population:** 6 patients with disfiguring local mandibular deformities -**Intervention:** PEEK patient-specific onlay implants (PSIs)	• CT evaluation over 6 months showed favourable soft and hard tissue changes
13	Knitschke M, Sonnabend S, Roller FC, Pons-Kühnemann J, Schmermund D, Attia S, Streckbein P, Howaldt H-P, Böttger S; 2022 [19]	A Retrospective analysis	-**Population:** Patients undergoing mandibular reconstruction with fibula free flap (FFF) -**Intervention:** Titanium laser-melted patient-specific implants (PSIs)	• OPG, CT, and CBCT evaluations assessed ossification at mandible–fibula and fibula–fibula junctions. Longer time period required; progression of subtotal ossification of FFF segments remains unclear
14	Yang HJ, Oh JH; 2022 [20]	Case series	-**Population:** Patients with mandibular contour defects -**Intervention:** 3D patient-specific titanium implants with three screw holes	• CT evaluation at 6-month follow-up showed good surgical accuracy and postoperative stability
15	Abbas SEM, ELKhashab MA.; 2021 [21]	Case report	-**Population:** Patient with soft tissue complication associated with implant-retained FPD -**Intervention:** 3D-printed patient-specific titanium implant	• Three-year follow-up showed stable outcomes; limitation noted in oral hygiene maintenance
16	Shaikh MQ, Nath SD, Akilan AA, et al.; 2021 [22]	In vitro study	**-Population/Procedure:** Digital oral anatomy data processed for design and fabrication of Ti-6Al-4 V maxillofacial implants using MF3 technology **-Intervention:** Patient-specific maxillofacial implant prototype	• Favours bone healing • Good osseointegration
17	Olate S, Uribe F, Huentequeo-Molina C, Goulart DR, Sigua-Rodriguez EA, Alister JP, 2021 [23]	Critical analysis	-**Population:** 21 patients undergoing facial surgery -**Intervention:** Stock implants vs. CAD/CAM-designed PEEK 3D patient-specific implants	• CBCT evaluation at 6-month follow-up assessed surgical time, intra- and post-operative complications. Outcomes were favourable, though limited by small sample size and short follow-up
18	Mommaerts MY, 2021 [24]	Questionnaire study	-**Population:** 21 patients evaluated through questionnaires and facial panel scoring before and after implantation -**Intervention:** Patient-specific 3D-printed titanium implants	• Six-month follow-up showed improved outcomes; however, the study was limited by low response rate, and retrospective design
19	SuojanenJ, Hodzic Z, Palotie T, Stoor P.; 2020 [25]	Case report	-**Population:** Patient with acromegaly presenting facial deformity and posterior open bite -**Intervention:** LeFort I and modified subcondylar osteotomies using CAD-CAM drill guides with patient-specific implants (PSIs)	• Lateral cephalometric and OPG evaluations over 22 months showed accurate jaw movement correction and stable outcomes
20	Wang, Y.-T., Chen, C.-H., Wang, P.-F., Chen, C.-T., & Lin, C.-L.; 2020 [26]	Finite element analysis	-**Population/Model:** Intact facial skeletal (IFS) model with ZMC fracture fixation -**Intervention:** Metal 3D-printed patient-specific repairing thin (PSRT) implant vs. two traditional miniplates	• CT-based biomechanical analysis under 250 N axial load showed effective fixation with PSRT implant compared to traditional plates
21	Mounir M, Abou-ElFetouh A, ElBeialy W, Mounir R., 2020 [27]	case series	-**Population:** 4 patients with segmental mandibular defects -**Intervention:** Patient-specific titanium implants (PSIs)	• OPG and CBCT evaluations over 3–5 years showed reconstruction outcomes but with a high failure rate as a key limitation
22	Murnan EJ, Christensen BJ.,2020 [28]	Retrospective cohort study	-**Population:** 32 patients treated with patient-specific PEEK implants -**Intervention:** PEEK implants placed adjacent to paranasal sinuses (56.3%); indications included malar depression (50%), orbital dystopia (46.9%), forehead/skull defects (21.9%), and mandibular contour deformities (6.2%); some patients had multiple indications	• Statistical analyses (Fisher exact, t-tests, multivariable logistic regression) confirmed effectiveness and applicability across varied craniofacial defects
23	Igelbrink S, Zanettini LMS, Bohner L, Kleinheinz J, Jung S,2020 [29]	Case report	-**Population:** Patient with aesthetic defect of unilateral hypoplastic mandible post-orthognathic surgery -**Intervention:** Two-piece titanium patient-specific implant	• CT and digital design with Geomagic Freeform guided fabrication; 1-year follow-up showed satisfactory aesthetic outcome
24	Wong WW, Martin MC, 2016 [30]	Case report	-**Population:** Patient with polyostotic juvenile ossifying fibroma -**Intervention:** Hemimandibulectomy reconstructed with fibular grafts fixed to mandibular plate; orbitomaxillectomy defects reconstructed with flaps and PSI titanium rim implant; nasal lining reconstructed with septal flaps	• Excellent facial contour and symmetry • At 7-month follow-up, reconstruction showed satisfactory functional and aesthetic outcomes
25	Staal F, Pluijmers B, Wolvius E, Koudstaal M.; 2016 [31]	Case series	-**Population:** Patients with unilateral underdeveloped mandible -**Intervention:** • **Case 1:** Mandibular distraction osteogenesis (DO) + Le Fort I osteotomy; Medpor implant • **Case 2:** Mandibular DO + Le Fort I osteotomy + coronoidectomy + condylar reconstruction with costochondral rib graft; PEEK implant	• 3D CT evaluation showed stable reconstruction; follow-up was 57 months (Case 1) and 32 months (Case 2)
26	Scolozzi P.; 2015 [32]	Case report	**-Population:** Patients with dentofacial deformities (*n* = 10) -**Intervention:** CAD/CAM PEEK patient-specific implants with surgical splints/cutting guides for maxillomandibular asymmetries (4 patients), customized distractors for severe maxillary deficiencies (3 patients), and custom implants for chin contouring/mandibular defects post-osteotomies (3 patients)	• No postoperative complications • CBCT evaluation at 1-year follow-up showed satisfactory functional and aesthetic outcomes

### Characteristics of included studies

#### Customization aspects

Among the 26 included articles, a key focus was on manufacturing methods and materials used for patient-specific implants (PSIs). Gugliotta and associates evaluated cranio-maxillofacial reconstruction using polyetheretherketone (PEEK) PSIs and reported excellent aesthetic and functional outcomes [7]. Similarly, Atef et al. studied soft and hard tissue changes following augmentation of mandibular defects using PEEK onlay PSIs and observed favourable aesthetic results [18]. Datarkar et al. (2024) performed an FEA analysis on a PSI with strut abutment interface to assess the stress concentration on the implant and surrounding bone in bilateral maxillectomy defect. The results of this study showed that the precise accuracy in stress concentration and improves the quality of life of patients [8]. Arvind et al., rehabilitated the maxilla with zygomatic and 3D-Manufactured Patient-Specific Implants in Mucormycosis Post-COVID-19 and showed efficient therapeutic outcomes. Hwang et al. proposed a surgical option that combined the microvascular fibula free flap with a 3D-bioprinted, patient-specific polycaprolactone (PCL) implant, a safe and simple novel method that produced the best functional and aesthetic results in mandibular reconstruction surgery for young patients with malignant tumours [9]. Ayhan M et al. used FEA analysis in a custom-made subperiosteal implant placed in the atrophic maxilla. This study aimed to investigate and compare the stress distribution, displacement, and bone loading of PSI and concluded that the stress distribution on implants subjected to mastication was significantly lower [14]. Yang et al. (2022), evaluated the surgical accuracy and postoperative stability of patient-specific titanium implants (PSTIs) for mandibular reconstruction, reporting high patient satisfaction with no postoperative complications [20]. Suojanen et al., reported the correction of facial deformity and posterior open bite with Le Fort I and modified subcondylar osteotomies in a patient affected by acromegaly with the help of CAD/CAM engineered patient-specific implants. The procedure resulted in excellent esthetic and functional outcomes. [25]. Zhen Yang et al., proposed a minimally invasive implant restoration which was customized with short titanium implants with wing retention. The design of the wings influenced the stress distribution and implant stability [12].

#### Surgical integration

Alwala et al., reported that computer-designed PSI offers higher accuracy and defect adaption, enhanced stability and more predictable outcomes [11]. Yang et al., 2022, also evaluated the post-operative stability of patient-specific titanium implants for mandibular defects. It was also found that more than 500 µm porosity increases osteoinduction in Ti implants fabricated by the SLM method, which may promote long-term stability of implants [20]. PSIs have shown excellent osseointegration between the implant and bone, irrespective of different materials and surgical techniques. Datarkar et al., 2024, reported that 100% osseointegration was achieved between the screws of the implant and the bone with a precise surgical technique [8].

#### Complications

Most of the studies included in Table 2 mentioned that customized implants improve the quality of life of patients by rehabilitating the defect with different PSI materials and manufacturing methods. Some studies reported the complications of PSI, which include soft tissue dehiscence and post-operative infection. It was reported that the overall patient satisfaction was high, with no long-term complications. Additional reported adverse outcomes include prosthesis failure in cases of high functional load and difficulties in maintaining oral hygiene, which may predispose patients to peri-implant infection.

#### Clinical outcome

PEEK PSIs, 3D-printed PSIs, and CAD/CAM PSIs have shown excellent survival rates in different studies, but factors like material properties, surgical technique, and patient-specific anatomy play a significant role. Most of the studies reported the good esthetic and functional outcomes regardless of the material and technique used. Patient-specific implants (PSIs) show favorable clinical outcomes for the atrophic maxilla, providing considerable aesthetic and functional improvements, with high implant stability and good prosthetic success rates reported in studies. Most patients reported high satisfaction with improved quality of life. Long-term follow-up data further support stable outcomes with minimal adverse events.

#### Follow- up duration

Patient-specific implants (PSIs) often involve a follow-up period to monitor healing, implant stability, and overall patient outcomes. The duration of this follow-up can vary, but it generally includes regular check-ups with imaging (like CT scans) to assess the implant's position and integration with the bone. Studies reported the follow-up period as 6 months,12 months,2.5 years which shows the reliability and durability to rehabilitate the defect with PSI with no long-term complications reported within the patients.

## Discussion

Considering the compiled evidence, several important considerations emerge regarding the design, application, and clinical integration of patient-specific implants.

### Rehabilitation aspect

Patient-specific implants (PSI) are a personalized approach to reconstructive and cosmetic surgery. This is especially important in maxillofacial surgery and prosthodontics, where restoring the complex three-dimensional (3D) contour can be challenging. In some cases, the optimum outcomes can only be obtained with implants that are custom created to meet a specific requirement [33]. Congenital facial disorders are associated with skeletal deficiencies and abnormalities of the face that are extremely difficult to reconstruct. To improve facial contour, certain procedures are indicated, such as osteotomies, bone distraction, and grafting. The results of these surgeries vary, depending on criteria such as graft survival, precision of bone manipulation, and healing [34]^.^ PSIs could also be utilized in the rehabilitation of unilateral defects of the face, fabricating implants using a mirror image of the opposite healthy front [35].

Patient-specific subperiosteal implants, fabricated using direct metal laser sintering (DMLS), have shown promising results in the rehabilitation of atrophic mandibular ridges in elderly patients. One study reported a particularly satisfactory implant fit and a 100% survival rate after a one-year follow-up, indicating the potential reliability of this approach in compromised anatomical conditions [36].

PSI has been used successfully to repair numerous facial structures, such as fractured orbital walls, the floor of the orbit, and to operate on other maxillofacial deformities [37, 38]. Surgically acquired defects following tumor treatment frequently present challenges in oral rehabilitation, influencing the quality of life of these individuals. Jehn et al. assessed the Oral Health Impact Profile (OHIP) in 12 patients who were treated with patient-specific implants for severe bone insufficiency. The study indicated that patient-specific dental implants, particularly when combined with fixed dentures, contribute to improved oral health-related quality of life (OHRQoL) in patients with significant bone deficits [39].

### Material aspect

Patient-specific implants (PSI) are often made from metals, polymers, and ceramics using additive manufacturing technology. Titanium has been considered as a preferred metal in the reconstruction of patient-specific implants (PSI) [37], and total mandibular replacement [15, 36]. Among ceramics, metallic oxides, calcium phosphate, and glass ceramics are often used. The materials used are the least toxic and are highly biocompatible [40].

With recent advancements in technology, Polyether ether ketone (PEEK) has emerged as a promising alloplastic material that could be employed as an alternative in the manufacture of patient-specific implants. [34]. Many studies reports the positive esthetic and functional outcome with PEEK PSI [7, 18]. One of the studies conducted by Rosenthal et al. reported a contamination incidence of 7.7% among 65 cases in the study with prostheses made of PEEK [41].

Ultra-high molecular weight polyethylene (UHMW-PE) is employed in the restoration of orbit or temporomandibular joints by fabricating PSIs using CAD-CAM technology. UHMW-PE has been shown to have a lower incidence of infection rate when compared to porous polyethylene (PPE) [42]. Hwang B et al. employed a Patient-Specific Polycaprolactone (PCL) Implant for mandibular reconstruction and reported no complications for over 6 years. It has also been reported to have an accurate and safe surgical results [15].

### Manufacturing aspect

Initially, the manufacturing of patient-specific implants was done using subtractive manufacturing techniques [34]. However, it was noted that a significant amount of material was wasted, and it failed to replicate complex anatomical shapes using computer numerical control (CNC). This paved the path for the development of patient-specific implants using CAD/CAM and 3D printing [11, 25].

The manufacturing technique used for the fabrication of patient-specific implants is ideally carried out by taking into consideration such as nature of the material, convenient technology, characteristics, processing time, post-processing accuracy, and surface quality [43].

### Limitations and future scope

The cost of patient-specific implants is considerably higher than that of pre-bent or standard implants, primarily due to the need for specialized software, advanced 3D printing technology, and a customized design workflow [44]. Unanticipated modifications to the surgical site, such as changes in resection margins, can impact the precision and adaptation of the PSI [45]. Although PSIs offer advantages in oncologic cases, tumor recurrence and radiation-related complications may limit their effectiveness. Combining PSIs with microvascular bone flaps can help restore optimal height and contour for mandibular rehabilitation [5]. Despite being custom designed for accuracy, PSIs may require intraoperative adjustments due to anatomical discrepancies or surgical constraints [45]. Although automation has enhanced procedural efficiency, surgical expertise remains essential for accurate PSI placement and effective management of complications. Though there are challenges, PSIs offer significant benefits in fit, precision, and operative efficiency in maxillofacial procedures [46].

The development of next-generation biomaterials with superior biocompatibility, durability, and resorption profiles is key to optimizing PSI outcomes [22]. Current research is investigating the use of PSIs as scaffolding platforms for tissue regeneration, with the aim of achieving more natural and durable reconstructive outcomes [47]. As 3D printing continues to advance, meeting regulatory requirements will be essential to ensure the safety and effectiveness of medical devices. In silico methods like finite element analysis allow preoperative evaluation of PSIs by simulating mechanical behavior, resorption, and bone growth. Optimization techniques can further personalize implant design to enhance surgical outcomes [48]. Emerging technologies such as bioprinting, in silico modeling, and tissue engineering are driving advances in personalized medicine. Bioprinting enables the fabrication of functional tissues, in silico modeling supports treatment planning, and tissue engineering aids tissue regeneration, collectively paving the way for customized therapies [49].

## Conclusion

Patient specific implants not only produce desirable outcomes but also significantly improve the quality of life of the individuals. However, limitations such as high cost, longer fabrication time, and risk of post-operative complications remain important considerations. Further improvements in CAD/CAM technology, will allow the fabrication of patient-specific implants at a considerably lower cost, simultaneously enhancing precision and overall results. Future research in bioprinting and tissue engineering may enable the development of PSIs using autologous tissue and biodegradable materials, further advancing personalized reconstruction.

## Supplementary Information

Supplementary Material 1: Supplementary file 1: Table S1: Search strategy for each electronic database and websites of relevant journals. Table S2: Summary of included studies reporting on the use of customized, patient-specific implants for intraoral and maxillofacial reconstruction.

## Data Availability

No datasets were generated or analysed during the current study.

## Abbreviations

PSI: Patient-specific implant
3D: Three dimensional
CAD-CAM: Computer aided Design and computer aided manufacturing
PEEK: Poly ether ether ketone
FEA: Finite Element Analysis
SLM: Selective Laser Melting
MRI: Magnetic resonance imaging
OPG: Orthopantomogram
CT: Computed tomography
