# Supplementary material for: Patient-specific implants for intraoral and maxillofacial reconstruction: a scoping review on customization and fabrication methods
Source: Maxillofac Plast Reconstr Surg. 2025 Oct 7;47(1):28. doi: 10.1186/s40902-025-00485-6 (PMC12504169; doi:10.1186/s40902-025-00485-6)
Supplement: Supplementary file 1 — Supplementary Material 1: Supplementary file 1: Table S1: Search strategy for each electronic database and websites of relevant journals. Table S2: Summary of included studies reporting on the use of customized, patient-specific implants for intraoral and maxillofacial reconstruction. [file 40902_2025_485_MOESM1_ESM.docx]

**Tables**

Table 1: Search strategy for each electronic database and websites of relevant journals.

| Database | Search terms | No of articles |
| --- | --- | --- |
| PubMed | "patient-specific implant"[Text Word] AND ("intraoral defect"[Text Word] OR (("mandible"[MeSH Terms] OR "mandibular"[Text Word]) AND ("abnormalities"[MeSH Subheading] OR "abnormalities"[All Fields] OR "defects"[All Fields] OR "defect"[All Fields] OR "defect s"[All Fields] OR "defected"[All Fields] OR "defective"[All Fields] OR "defectively"[All Fields] OR "defectives"[All Fields])) OR "maxillary defect"[Text Word] OR ("maxillofacial"[All Fields] AND ("abnormalities"[MeSH Subheading] OR "abnormalities"[All Fields] OR "defects"[All Fields] OR "defect"[All Fields] OR "defect s"[All Fields] OR "defected"[All Fields] OR "defective"[All Fields] OR "defectively"[All Fields] OR "defectives"[All Fields]))) AND ("computer-aided design"[MeSH Terms] OR "cad-cam"[Text Word] OR ("printing, three dimensional"[MeSH Terms] OR "3d printing"[Text Word]) OR (("addition"[All Fields] OR "additional"[All Fields] OR "additions"[All Fields] OR "additive"[All Fields] OR "additively"[All Fields] OR "additives"[All Fields] OR "additivities"[All Fields] OR "additivity"[All Fields]) AND ("manufacturability"[All Fields] OR "manufacturable"[All Fields] OR "manufacture"[All Fields] OR "manufacture s"[All Fields] OR "manufactured"[All Fields] OR "manufacturer"[All Fields] OR "manufacturer s"[All Fields] OR "manufacturers"[All Fields] OR "manufactures"[All Fields] OR "manufacturing"[All Fields]))) | 28 |
| SCOPUS | TITLE-ABS-KEY("patient-specific implant") AND (TITLE-ABS-KEY("intraoral defect") OR ((INDEXTERMS(mandible) OR TITLE-ABS-KEY(mandibular)) AND (INDEXTERMS(abnormalities) OR ALL(abnormalities) OR ALL(defects) OR ALL(defect) OR ALL("defect s") OR ALL(defected) OR ALL(defective) OR ALL(defectively) OR ALL(defectives))) OR TITLE-ABS-KEY("maxillary defect") OR (ALL(maxillofacial) AND (INDEXTERMS(abnormalities) OR ALL(abnormalities) OR ALL(defects) OR ALL(defect) OR ALL("defect s") OR ALL(defected) OR ALL(defective) OR ALL(defectively) OR ALL(defectives)))) AND (INDEXTERMS("computer-aided design") OR TITLE-ABS-KEY(cad-cam) OR (INDEXTERMS("printing, three dimensional") OR TITLE-ABS-KEY("3d printing")) OR ((ALL(addition) OR ALL(additional) OR ALL(additions) OR ALL(additive) OR ALL(additively) OR ALL(additives) OR ALL(additivities) OR ALL(additivity)) AND (ALL(manufacturability) OR ALL(manufacturable) OR ALL(manufacture) OR ALL("manufacture s") OR ALL(manufactured) OR ALL(manufacturer) OR ALL("manufacturer s") OR ALL(manufacturers) OR ALL(manufactures) OR ALL(manufacturing)))) | 336 |
| Web of Science | "patient-specific implant" AND ("intraoral defect" OR ((mandible OR mandibular) AND (abnormalities OR abnormalities OR defects OR defect OR "defect s" OR defected OR defective OR defectively OR defectives)) OR "maxillary defect" OR (maxillofacial AND (abnormalities OR abnormalities OR defects OR defect OR "defect s" OR defected OR defective OR defectively OR defectives))) AND ("computer-aided design" OR cad-cam OR ("printing, three dimensional" OR "3d printing") OR ((addition OR additional OR additions OR additive OR additively OR additives OR additivities OR additivity) AND (manufacturability OR manufacturable OR manufacture OR "manufacture s" OR manufactured OR manufacturer OR "manufacturer s" OR manufacturers OR manufactures OR manufacturing))) | 45 |
| COCHRANE | "patient-specific implant":ti,ab,kw AND ("intraoral defect":ti,ab,kw OR (([mh mandible] OR mandibular:ti,ab,kw) AND ([mh /abnormalities] OR abnormalities OR defects OR defect OR "defect s" OR defected OR defective OR defectively OR defectives)) OR "maxillary defect":ti,ab,kw OR (maxillofacial AND ([mh /abnormalities] OR abnormalities OR defects OR defect OR "defect s" OR defected OR defective OR defectively OR defectives))) AND ([mh "computer-aided design"] OR cad-cam:ti,ab,kw OR ([mh "printing, three dimensional"] OR "3d printing":ti,ab,kw) OR ((addition OR additional OR additions OR additive OR additively OR additives OR additivities OR additivity) AND (manufacturability OR manufacturable OR manufacture OR "manufacture s" OR manufactured OR manufacturer OR "manufacturer s" OR manufacturers OR manufactures OR manufacturing))) | 7 |
| Google Scholar | "patient-specific implant" AND ("intraoral defect" OR "mandibular defect" OR "mandible abnormalities" OR "maxillary defect" OR "maxillofacial defect") AND ("computer-aided design" OR "CAD-CAM" OR "3D printing" OR "additive manufacturing") | 229 |

Table 2: Summary of included studies reporting on the use of customized, patient-specific implants for intraoral and maxillofacial reconstruction

| Sr no: | Title | Authors & Year of Publication | Type of research | Intervention | Results |
| --- | --- | --- | --- | --- | --- |
| 1. | Cranio-Maxillo-Facial Reconstruction with Polyetheretherketone Patient-Specific Implants: Aesthetic and Functional Outcomes ^[7]^ | Gugliotta Y, Zavattero E, Ramieri G, Borbon C, Gerbino G; 2024 | Case series | - Cranio-maxillo-facial reconstructions of 45 patients. - Implants placed: PEEK PSI - Parameters assessed: morpho-functional outcome through a modified Katsuragy Scale, the Visual Analogue Scale (VAS) for pain, and four FACE-Q\|Aestheticsc scales. - Duration of treatment: 5years after the resection surgery - Follow up:12 months | - No prosthesis dislocation, rupture, or long-term infection - Complication: post operative infection |
| 2. | A 3-Dimensional finite element analysis of patient-specific implant (with strut abutments) interface on stress concentration on the implant and surrounding bone in bilateral maxillary deformities ^[8]^ | Datarkar, Abhay N.; Pardiwala, Arwa F.; Relan, Priyanka; Daware, Surendra; Gadve, Vandana; Deshpande, Archana; Ghormade, Ashlesha; 2024 | Finite element analysis | - Patients with bilateral maxillectomy defect - Parameters assessed: physiological and pathological loading conditions, failure and fatigue of the implant structure, osseointegration, stress shielding of bone. - Implants placed: PSI with strut abutments - Investigations: CT | - 100% osseointegration between the screws of the implant and the bone. - Improved accuracy in stress distribution - Improves the quality of life of patients. |
| 3. | Integrative Approach to Maxillary Reconstruction: Assessment of Proficiency of Zygomatic and 3D-Manufactured Patient-Specific Implants in Mucormycosis Post-COVID-19^[9]^ | Arvind U.D, Gaddipati, R., Alwala, A.M.; 2024 | In vivo | - 10 patients underwent surgical and prosthetic maxillary reconstruction - Implants placed: zygomatic implants and 3D-manufactured patient-specific implants. - Parameters assessed: pain, implant exposure, infection, wound dehiscence, implant fit, post-operative surgical rating scale, oro-antral communication, facial edema, and orbital status were evaluated at baseline, 6 months, and 12 months post-surgery. - Limitations: Less number of sample size | - efficient therapeutic outcomes for maxillary reconstruction - Good patient satisfaction |
| 4. | Validation of Low-Cost Patient Specific Implant Design Using Finite Element Analysis (FEA) for Reconstruction of Segmental Mandibular Defects: A Case Report and Literature Review. ^[10]^ | Chakravarthy C, Patil RS, Wagdargi S, et al,2024 | Case report | - Patient with segmental mandibular defect. - Implants placed: customized computer-designed patient-specific implants (PSIs) - Parameters assessed: aesthetics and the individual clinical situation. - Investigations: CT, OPG - Follow up: 2.5 years | - good facial symmetry - normal mouth opening - no TMJ problems - Good accuracy and stability of PSI. |
| 5. | Analysis of the Effectiveness of 3D Printed Patient-Specific Implants for Reconstruction of Maxillary Defect Secondary to Mucormycosis ^[11]^ | Alwala A.M., Ramesh, K., Swayampakula, H; 2023 | In vivo study | - 20 patients with surgical and prosthetic reconstruction of the maxilla. - parameters assessed: pain, implant exposure, infection, wound dehiscence, fit of implant, postoperative surgical rating scale, and patient experience evaluation rating scale at baseline,3, 6 and 12 months. - Implants placed: 3D printed titanium subperiosteal implants. - Follow up: 1 year - Limitation: Smaller sample size. | - Good result which needs more sample size - longer follow-up period |
| 6. | Biomechanical evaluation of custom-made short implants with wing retention applied in severe atrophic maxillary posterior region restoration: A three-dimensional finite element analysis ^[12]^ | Yang Z, Zhang J, Xu Z, Liu X, Yang J, Tan J; 2023 | Finite Element Analysis | - Patients with severe atrophic posterior maxilla - Parameters assessed: position, structure, and spread area of the wings fixture. - Investigation: CBCT - Implants: Short patient specific implants. | - The planar form can better disperse the stress. - By adjusting the cusp slope to reduce the influence of lateral force, short implants with planar wing fixtures can be used safely. |
| 7. | Comparison of two types of patient specific implants (PSI) and quad zygoma implant (QZI) for rehabilitation of post-COVID maxillary mucormycosis defect: Finite Element Analysis ^[13]^ | Manekar VS, Datarkar AN, Ghormode A, Daware S, Pandilwar P, Sapkal P.; 2023 | Finite Element Analysis | - Patient with post-covid maxillary mucormycosis with unilateral /bilateral defect - Parameters assessed: validate and compare the biomechanical benefit of the PSI struts (PSI 1), PSI screw retained (PSI 2), Quad zygoma implant (QZI) - Investigation: CT - Implants placed: CAD/CAM PSI, quad zygoma implant | - PSI 1, PSI 2 and QZI showed a good resistance to displacement. - The stress and strain values are low and acceptable. - QZI shows more stress in the anterior region. |
| 8. | Custom-made Subperiosteal Implants: A Finite Element Analysis on Monoblock and Dual Implant Systems in Atrophic Maxilla ^[14]^ | Ayhan M, Cankaya AB.; 2023 | Finite Element Analysis | - Patients with insufficient bone tissue for conventional implant treatment - Parameters assessed: Two different models were produced: a monoblock that covered the entire maxillary bone and a dual implant system where two mirror-imaged implants covered the left and right halves of the maxillary bone separately. - residual stress values and displacement values formed on the implant models and jawbone models were calculated separately. - Investigations: computed tomography (CT) scans. - Implants placed: 3D Customized subperiosteal implant designs | - The stresses formed on implants that are under the mastication forces were lower than the yield strength of the selected material, indicating that plastic deformation would not occur under static load. - Reduction in stress in dual implant geometry |
| 9. | Long-Term Follow-Up of a Novel Surgical Option Combining Fibula Free Flap and 3D-Bioprinted, Patient-Specific Polycaprolactone (PCL) Implant for Mandible Reconstruction. ^[15]^ | Hwang B Y, Noh K, Lee JW., et al, 2023 | Case report | - Patient with osteosarcoma of the left mandible body. - Surgical intervention   -segmental mandibulectomy  - rehabilitate with osteocutaneous fibula free flap and implant.   - Implants: 3D- bio printed PCL implant. - Investigations: CT, MRI - Follow up: CT: 2 weeks, once in every year for 6 years. MRI: 5,11,40 months and   4 .5 years   - Limitations: Expensive, time consuming | - No complications for over 6 years. - accurate and safe surgical results. - Good esthetic and functional outcome. |
| 10. | Rehabilitation of patient using a patient-specific implant with bar-retained maxillary obturator and orbital prosthesis post operated rhino-orbital mucormycosis ^[16]^ | V jain, Vaibhav; Nagori, Shakeel. ,2023 | Case report | - COVID patient was diagnosed with mucormycosis and underwent right orbital exenteration & maxillectomy. - Investigation: CT - Implants placed: 3D printed PSI. - Follow up: up to 74 months | - Good esthetic outcome - Improved Patient satisfaction - Excellent implant stability |
| 11. | Comparison of conventional and digital workflow for dental rehabilitation with a novel patient-specific framework implant system: an experimental dataset evaluation ^[17]^ | Simon Spalthoff S., Borrmann, M., Jehn, P.; 2022 | In vitro study | - Procedure: 25 Digital datasets of temporary prostheses fabricated on virtually constructed edentulous maxillae in two ways: one dataset comprised prostheses fabricated conventionally and then scanned using a model scanner, whereas the other dataset was designed virtually using standardized virtual dental arches. - Parameters assessed: -efficiency of digital workflow - Comparing the accuracy of prosthetic teeth positioning digitally and conventionally - Investigation: CT - Implants placed: Patient specific maxillofacial implant. | - The conventional design pathway was more accurate than the digital one. - patient satisfaction with esthetics. |
| 12. | Polyetheretherketone patient-specific implants (PPSI) for the reconstruction of two different mandibular contour deformities ^[18]^ | Atef M Mounir M, Shawky M, Mounir S, Gibaly A,  2022 | In vivo | - 6 patients with disfiguring local mandibular deformities - Implant placed: PEEK patient-specific onlay implants (PSI), - Parameters assessed: soft and hard tissue changes - Follow up: 6-month - Investigation: CT | - Good esthetic outcome. |
| 13. | Osseous Union after Mandible Reconstruction with Fibula Free Flap Using Manually Bent Plates vs. Patient-Specific Implants: A Retrospective Analysis of 89 Patients. ^[19]^ | Knitschke M, Sonnabend S, Roller FC, Pons-Kühnemann J, Schmermund D, Attia S, Streckbein P, Howaldt H-P, Böttger S; 2022 | A Retrospective analysis | - Evaluating the ossification of junctions between mandible and fibula and between osteotomized fibula free flap (FFF) segments. - Investigations: Panoramic radiograph (OPT), computed tomography (CT) scans, or cone-beam CTs (CBCT) - Implants placed: Titanium laser melted PSI. - Limitations: Longer time period, progression of subtotal ossification of free flap segments remains unclear | - PSI implants allow highly accurate poly-segmental, and therefore more complex shaping and molding of a fibula free flap - Incomplete ossification more frequently in PSI. - Improves fibula-jawbone healing - Low post-operative complications - Improves physiologic bone remodelling in PSI system. |
| 14. | Reconstruction of Mandibular Contour Defect Using Patient-Specific Titanium Implant Manufactured by Selective Laser Melting Method ^[20]^ | Yang HJ, Oh JH; 2022 | Case series | Patients with mandibular contour defects   - Implant placed: 3D- Patient-specific titanium implant - Procedure: 3 screw holes were formed. Patient-specific titanium implant. - Surgical accuracy and postoperative stability were evaluated - Investigations: computed tomography (CT), - Follow up: 6-month | - Improved patient satisfaction - no complications - Improved surgical accuracy - good postoperative stability |
| 15. | Case Report Soft Tissue Dehiscence Associated with a Titanium Patient-Specific Implant: A Prosthetic Solution as an Alternative to Soft Tissue Grafting ^[21]^ | Abbas SEM, ELKhashab MA.; 2021 | Case report | - Patient with soft tissue complication with implant retained FPD. - Implant placed: 3D printed patient-specific titanium implant. - Follow up: 3 years - Limitation: oral hygiene maintenance | - significant satisfaction with the aesthetics and function of the prosthesis. |
| 16. | Investigation of Patient-Specific Maxillofacial Implant Prototype Development by Metal Fused Filament Fabrication (MF3) of Ti-6Al-4V. ^[22]^ | Shaikh MQ, Nath SD, Akilan AA, et al; 2021 | In vitro study | - Procedure: processing the digital data of the patient’s oral anatomy to design development and fabrication of Ti-6Al-4V maxillofacial implants using metal fused filament fabrication (MF3) technology. - Parameters assessed: the feasibility of metal fused filament fabrication (MF^3^) to manufacture patient-specific maxillofacial implants was investigated - Investigation: CBCT - Implants: Patient specific maxillofacial implant prototype | - Favours bone healing - Good osseointegration. |
| 17. | Mandibular Angle Contouring Using Porous Polyethylene Stock or PEEK-based Patient Specific Implants. A Critical Analysis ^[23]^ | Olate S, Uribe F, Huentequeo-Molina C, Goulart DR, Sigua-Rodriguez EA, Alister JP, 2021 | Critical analysis | - 21 Patients who were submitted to facial surgery - Implants placed: stock implant or PEEK 3D patient specific implants created with CAD/CAM technology - Parameters assessed:   Surgical time, intra-operative and post-operative complications.   - Investigation: CBCT - Follow up: 6 months - Limitations: small sample size, minimal follow up | - The 3D printed implants had greater levels of facial symmetry than the stock implants. - Low complication rate. |
| 18. | Patient- and clinician-reported outcomes of lower jaw contouring using patient-specific 3D-printed titanium implants ^[24]^ | Mommaerts MY, 2021 | Questionnaire study | - Questionnaires sent to 21 patients and panel scores given to pictures of patients’ faces, before and after implantation. - Implants used: Patient specific 3D-printed titanium implants - Follow up: 6 months - Limitation:  1. Low response rate 2. Study was retrospective in nature and single- authored. | - Adverse effects resulting from jawline contouring with patient-specific 3D-printed titanium alloy implants were low. - minimal harm and complications. |
| 19. | CAD/CAM Engineered Patient-Specific Implants as a Reposition Device in Le Fort I and Modified Subcondylar Osteotomies: Case Report of Facial Deformity Correction in Acromegaly. ^[25]^ | SuojanenJ, Hodzic Z, Palotie T, Stoor P.; 2020 | Case report | - Correction of facial deformity and posterior open bite with LeFort 1 and modified sub condylar osteotomies in a patient affected by acromegaly. - Surgical correction: CAD-CAM generated drill guides were used to perform osteotomies and segment removal ensuring the planned movement of the jaws and position of PSI. - Investigations: lateral cephalometric, OPG - Implant placed: patient-specific implants (PSIs) - Follow up: 22 months | - good functional and aesthetic outcome. |
| 20. | Design of a Metal 3D Printing Patient-Specific Repairing Thin (PSRT) Implant for Zygomaticomaxillary Complex Bone Fracture Based on Buttress Theory Using Finite Element Analysis ^[26]^ | Wang, Y.-T., Chen, C.-H., Wang, P.-F., Chen, C.-T., & Lin, C.-L.; 2020 | Finite element analysis | - An intact facial skeletal (IFS) model was constructed. - Parameters assessed: biomechanical analysis on a ZMC fracture fixation with the PSRT implant and two traditional mini plates under uniform axial loads applied on posterior teeth with 250 N. - Investigation: CT - Implant placed: Metal 3D printed patient specific repairing thin implant | - Small stress variations between the IFS model and repairing with a PSRT implant were found in the frontal and zygomatic process. - Comparatively, large stress variations with different distributions between the IFS model and mini-plate models were found at the corresponding areas. |
| 21. | Patient-specific alloplastic endoprosthesis for reconstruction of the mandible following segmental resection: A case series ^[27]^ | Mounir M, Abou-ElFetouh A, ElBeialy W, Mounir R., 2020 | case series | - 4 Patients with segmental mandibular defects - Implants placed: patient-specific titanium implant (PSI) - Investigations: OPG, CBCT - Follow-up: 3-5 years - Limitations: High failure rate | - good results in terms of occlusion, masticatory function, mouth opening as well as pleasing facial aesthetics. |
| 22. | Risk Factors for Postoperative Inflammatory Complications After Maxillofacial Reconstruction Using Polyether-Ether-Ketone Implants ^[28]^ | Murnan EJ, Christensen BJ. ,2020 | Retrospective cohort study | - 32 Patients with patient-specific PEEK implants - The PEEK implant was placed adjacent to the paranasal sinuses in 56.3% of patients. The indication for use was malar depression in 50.0%, orbital dystopia in 46.9%, forehead or skull defects in 21.9%, and mandibular contour deformities in 6.2%; 8 patients had more than 1 indication. - Statistical analysis: Fisher exact tests, t tests, and multivariable logistic regression analysis where appropriate. | - Tobacco use, the presence of an intraoral incision, and the presence of multiple incisions were all associated with POICs. |
| 23. | Three-Dimensional Planning of the Mandibular Margin in Hemifacial Microsomia Using a Printed Patient-Specific Implant ^[29]^ | Igelbrink S, Zanettini LMS, Bohner L, Kleinheinz J, Jung S ,2020 | Case report | - Patient with esthetic defect of the unilateral hypoplastic mandible after completion of the orthognathic surgery attended for consultation. - Investigation: CT scan and geomagic freeform software - Implant used: 2-piece titanium implant - Follow up: 1 year after the surgery. | - increase in bony symmetry. - No postoperative pain or trismus - Better wound healing |
| 24. | Reconstruction of Extended Orbitomaxillectomy and Hemimandibulectomy Defects with Fibula Flaps and Patient-Specific Implants ^[30]^ | Wong WW, Martin MC, 2016 | Case report | - Resection of the polyostotic juvenile ossifying fibroma. - Procedure: Reconstruction of the hemimandibulectomy defect with fibular grafts. Followed by the fibular bone segments were fixated to the mandibular plate. - Reconstruction of orbitomaxillectomy defects with flaps followed by placement of PSI titanium rim implant. - Reconstruction of nasal lining with nasal septal flaps. - Follow up period: 7 months post-operatively | - Excellent facial contour and symmetry. - No recurrence of infection. |
| 25. | Patient-Specific Implant for Residual Facial Asymmetry following Orthognathic Surgery in Unilateral Craniofacial Microsomia. ^[31]^ | Staal F, Pluijmers B, Wolvius E, Koudstaal M.; 2016 | Case series | Patients with Unilateral Underdeveloped Mandible  **Case 1:**  Surgical corrections – Distraction osteogenesis (DO) of the mandible and a Le Fort I osteotomy combined with an osteotomy of the mandible  Investigation - 3D CT scan  Implants placed – Medpor  Follow-up time – 57 months  **Case 2:**  Surgical corrections - mandibular DO, reconstruction of the right mandibular condyle with a costochondral rib graft, a Le Fort I osteotomy combined with DO of the mandible, and a coronoidectomy on the right side  Investigation - 3D CT scan  Implants placed – PEEK implant   - Follow-up time – 32 months | - Good aesthetic outcome - No complication reported |
| 26. | Computer-aided design and computer-aided modelling (CAD/CAM) generated surgical splints, cutting guides and custom-made implants: Which indications in orthognathic surgery? ^[32]^ | Scolozzi P.; 2015 | Case report | - Patients with dentofacial deformities - **Procedure**   -Four patients had surgical splints and cutting guides for correction of maxillomandibular asymmetries  -Three had surgical cutting guides and customized internal distractors for correction of severe maxillary deficiencies  - Three had custom-made implants for additional chin contouring and/or mandibular defects following bimaxillary osteotomies and sliding genioplasty.   - Investigation: CBCT - Implants placed: CAD/CAM PEEK psi - Follow up: 1 year | - Good esthetic results - Good patient satisfaction - No postoperative complications. |
